# Ultrasound guidance for bedside placement of peripherally inserted central catheter in pediatric patients

**DOI:** 10.1186/2036-7902-4-S1-A16

**Published:** 2012-12-18

**Authors:** Juan José Menéndez Suso

**Affiliations:** 1 Pediatric Critical Care Department, La Paz Universitary Hospital, Madrid, Spain

## Background

Studies conducted in adults have revealed that ultrasound (US) guidance for peripherally inserted central catheter (PICC) placement may improve success rate and reduce procedural complications. However, this is still not sufficiently studied in children.

## Objective

To study the safety and efficacy of US-guidance for bedside PICC placement in children.

## Patients and methods

Prospective observational study in which 50 US-guided PICC placement attempts are analyzed. Patient clinical data, procedural details, and infectious and thrombotic complications of the catheters are described.

## Results

Median age and weight of the patients were 55 months (7-288) and 15 kg (3.2-80), respectively. The veins selected for PICC placement were basilic vein in 73% patients, brachial vein in 14.5%, cephalic vein in 6.3% and external yugular vein in 6.2%. Intravenous sedo-analgesia was administered in 93% of the patients. Successful PICC placement was achieved in 96% of attempts. Success rate was 42% in the first attempt, 58% in the second, and 79% in the third. Procedural complication rate was very low (8%), with moderate local hemorrhage and accidental arterial puncture incidence of 6% and 2%, respectively. The median time spent on the procedure was 28 minutes (15-90). The median cannulation time was 3.5 minutes (0.5-60). Median PICC dwell time was 17 days (4-59). Central line-associated bloodstream infection (CLABSI) was suspected in three cases but it was finally not confirmed in any case. Weekly echo-doppler exploration of the cannulated veins detected superficial vein thrombosis in 6.3% of the patients and deep vein thrombosis in 2%. No patients showed clinical signs of venous thrombosis.

## Conclusion

Ultrasound-guided PICC cannulation is safe, rapid, and has a high success rate in children.
